# Farmers’ perception of termites in agriculture production and their indigenous utilization in Northwest Benin

**DOI:** 10.1186/s13002-017-0187-2

**Published:** 2017-11-21

**Authors:** Laura Estelle Yêyinou Loko, Azize Orobiyi, Paterne Agre, Alexandre Dansi, Manuele Tamò, Yves Roisin

**Affiliations:** 1 Laboratory of Applied Entomology, Faculty of Sciences and Technology of Dassa, National University of Sciences, Technologies, Engineering and Mathematics of Abomey, BP 14 Dassa, Benin; 2 International Institute of Tropical Agriculture (IITA), PMB 5320, Ibadan, Oyo State Nigeria; 3 Laboratory of Biotechnology, Genetic Resources and Plant and Animal Breeding (BIORAVE), Faculty of Sciences and Technology of Dassa, BP 14 Dassa, Benin; 4 International Institute of Tropical Agriculture, 08 BP 0932, Cotonou, Benin; 5 Evolutionary Biology and Ecology, Université Libre de Bruxelles, Brussels, Belgium

**Keywords:** Management, Pest, Taxonomy, Termites, Usages, Vernacular nomenclature

## Abstract

**Background:**

Although termites are considered as agricultural pests, they play an important role in maintaining the ecosystem. Therefore, it matters to investigate the farmers’ perception of the impacts of the termites on the agriculture and their indigenous utilization.

**Methods:**

A semi-structured questionnaire was used to interview 94 farmers through 10 villages of Atacora department, in the northwestern region of Benin, to obtain information for the development of successful strategies of termite management and conservation. Their perceptions on the importance and management of termites along with the indigenous nomenclature and utilization of termite mounds were assessed. Termite species identified by farmers were collected and preserved in 80% alcohol for identification.

**Results:**

Eight crops were identified by farmers as susceptible to termites with maize, sorghum, and yam as being the most susceptible. According to farmers, the susceptibility to termites of these crops is due to their high-water content and sweet taste. A total of 27 vernacular names of termites were recorded corresponding to 10 species, *Amitermes evuncifer*, *Macrotermes subhyalinus*, and *Trinervitermes oeconomus* being the most damaging termite species. All the names given to termite species had a meaning. The drought was identified by farmers as the main factor favouring termite attacks. Demolition of termite mounds in the fields was the most commonly reported control method. Salt and other pesticides were commonly used by farmers to protect stored farm products. The lack of effective control methods is the main constraint for termite management. In northwestern Benin, farmers reported different purpose utilizations of termite mounds and termites.

**Conclusions:**

The study has shown that farmers perceived termites as pests of several agricultural crops and apply various indigenous control practices whose efficiency need to be verified. Utilization of termites and termite mound soil as food and medicinal resources underlines the need for a more focused approach to termite control for the conservation of non-pest termite species. The sensitization of farmers on the importance of termites as well as the development of an integrated control method to combat termite pests proved necessary.

## Background

Termites are social insects filling many ecological functions, especially in tropical ecosystems [1]. They play an important role in soil fertilization [2, 3], bioturbation and soil formation [4, 5], decomposition of organic matter [6, 7], and vegetation growth and diversity [3, 8]. However, termites are best known as pests, which cause severe damage to homes and agricultural products [9]. Of the more than 2600 described species of termites, only a few hundred are known as pests of food crops in Africa such as cereals [10–12], roots and tubers [13–16], legumes [17, 18], and fruit trees [19]. In northwest Benin, for instance, termites are highly voracious and destructive and cause substantial damage to agricultural products. The harvest losses caused by termites can be enormous, in the order of 20 to 45% [20]. Despite the huge amounts of damage to crops, very little information is known on farmers’ perceptions of termite pests and their management practices [21]. The first step towards the development of successful pest management strategies adapted to farmers’ needs is an understanding of farmers’ perceptions of the pests and their control methods [22, 23].

In Benin, apart from the previous studies conducted by Loko et al. [16] on farmers’ knowledge and perceptions of termites in yam (*Dioscorea* spp.) and by Togola et al. [11] on specific diversity and damage of termites on upland rice, few information were documented on termites in view of the antagonistic roles they can play in agriculture systems [16]. Hence, it is a priority to determine the perception of farmers on the importance of termites in agriculture. Such information is necessary for the formulation of a good pest management strategy [24]. Since sustainable termite management includes conservation of non-pest termite species and the utilization of termites and associated resources [25], it is important to document the indigenous utilizations of termites and termite mounds by farmers. Furthermore, information about indigenous perception of termite taxonomy is scarce in the literature, indicating the need for appropriate documentation of such invaluable information from a wide range of ethnic groups [26]. The objective of the present study was to determine farmers’ perception on the importance of termites as pests, indigenous nomenclature and taxonomy of termites, indigenous practices of termite control, and indigenous utilization of termites and termite mounds in northwestern Benin.

## Methods

### The study area and site selection

**Fig. 1:**
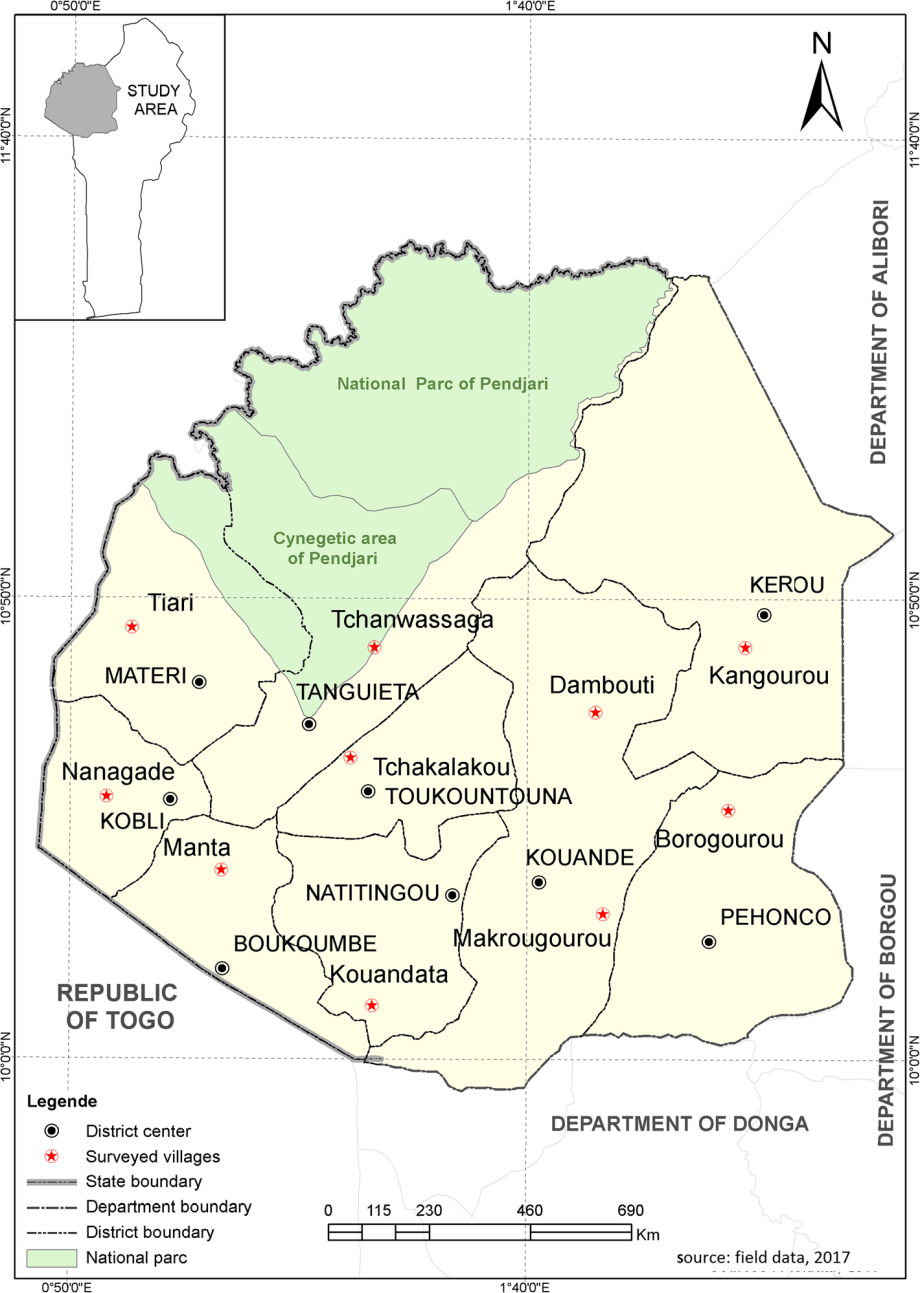
Map of Atacora department showing the geographical position of the surveyed villages

This study was conducted in Atacora department, located in northwestern Benin, and included nine districts: Boukoumbé, Kobli, Toukountouna, Kérou, Kouandé, Matéri, Natitingou, Péhonco, and Tanguiéta (Fig. 1). The region is characterized by the Atacora mountain chain, with altitudes varying from 400 m in the south to 650 m in the north. Atacora department has a sub-equatorial-type climate with only one dry season (November–March) and only one rainy season (April–October). The annual mean rainfall usually ranges from 800 to 1300 mm and the mean monthly temperature varies between 22 and 33 °C [27]. The main ethnic groups in Atacora department are Bariba, Berba or Biali, Ditamari, M’bermin or Gnindé, Waama, and Fulani or Peulh. The major means of survival for the local population is through agricultural crop farming, except for the Fulani ethnic group who major in animal husbandry. In this region, agricultural production is carried out by non-mechanized farms, which are dependent on human energy and still use relatively few inputs. The major crops grown include cereals (mainly maize and, to a lesser extent, sorghum, millet, and rice), roots and tubers (yams and cassava), and legumes (cowpea, beans, and voandzou). For a sufficient coverage study area, 10 villages were randomly selected and surveyed through the nine districts (Fig. 1).

### Data collection

The survey was implemented in each of the randomly selected villages through the application of participatory research appraisal tools and techniques, such as individual interviews and direct field observations using an interviewer-administered questionnaire according to Loko et al. [16]. Within villages, 6–10 households were randomly selected for individual interviews using the transect method described by Dansi et al. [28]. Because of the different ethnic groups involved, a translator or interpreter was locally recruited in each village to facilitate discussions and exchanges with farmers, following Loko et al. [29]. In each household, the interviewee was selected by mutual agreement with the hosting couple according to Christinck et al. [30]. A total of 94 households were interviewed. Data collected included socioeconomic data (age, gender, educational level, experience, and household size of the interviewees), farmers’ perception of importance of termites as pest (species crops susceptible to termites, reason of preference of termites for some crops, factors favouring the destructive behaviour of termites on crops, pest species, diversity of termite pest species, importance of damage), indigenous nomenclature and taxonomy of termites, indigenous practices of termite control, and indigenous utilization of termite mound soil and termites. When farmers identified a species of termite pest, they were asked to provide information on the crops they attack and to evaluate the damage caused.

### Collection and identification of termite species

Foraging soldiers and workers of all termite species identified by farmers were collected and preserved in 80% alcohol. Identification was conducted at the Laboratory of Evolutionary Biology and Ecology at the Université Libre de Bruxelles. The identification was based on the soldier caste. In the laboratory, the specimens were identified to species level by using various standard determination keys developed by Sands [31–34], Bouillon and Mathot [35, 36], Ruelle [37], and Williams [38].

### Statistical analysis

For all data, descriptive statistics (frequencies, percentages, means, and standard deviations) were calculated. Statistical analysis was performed using the Statistical Package for Social Sciences (IBM SPSS version 23.0).

## Results

### Sociodemographic characteristics of surveyed households

Eighty-eight percent of the surveyed households are male-headed with only 11.7% of the households being female-headed. The educational background of the household heads showed that 75.5% of them were illiterate with no formal education. A total of 20 household heads (21.3%) had basic education (primary school level), whereas two household heads (2.1%) received secondary education and only one (1.1%) household head had university level. The mean household heads’ age was 44.9 years, with a group range between 20 and 72 years of age (Table 1). The household heads have an average of 33.9 years of farming experience. Family size varied between 2 and 24 individuals, with an average of 8.5 individuals per household. The average land size owned by a household was 2.4 ha, and more than half of the surveyed household heads (62.8%) had 0.5–3 ha. Several ethnic groups were represented; the majority were Bariba (25.6%), Ditamari (21.3%), and Waama (21.3%), followed by Biali (10.6%), M’bermin (10.6%), and Peulh (10.6%).

**Table 1:** Sociodemographic characteristics of surveyed households in the study area (*n* = 94)

Demographic characteristics	Number of farmers	Percentage	Mean ± SE
Level of education			
No formal education	71	75.5	
Primary	20	21.3	
Secondary	2	2.1	
University	1	1.1	
Age (years)			
20–39	34	36.2	44.9 ± 1.1
40–49	28	29.8	
50–59	20	21.3	
60–72	12	12.7	
Gender			
Female	11	11.7	
Male	83	88.3	
Experience (years)			
7–25	25	26.6	33.9 ± 1.2
26–44	47	50.0	
45–62	22	23.4	
Household size			
2–10	68	72.3	8.5 ± 0.4
11–19	24	25.6	
20–24	2	2.1	
Land size			
0.5–3	59	62.8	
3–5	33	35.1	2.4 ± 0.1
5–10	2	2.1	

*n* number of interviewed household heads, *SE* standard error of the mean

### Farmers’ perception of termites as pests

**Fig. 2:**
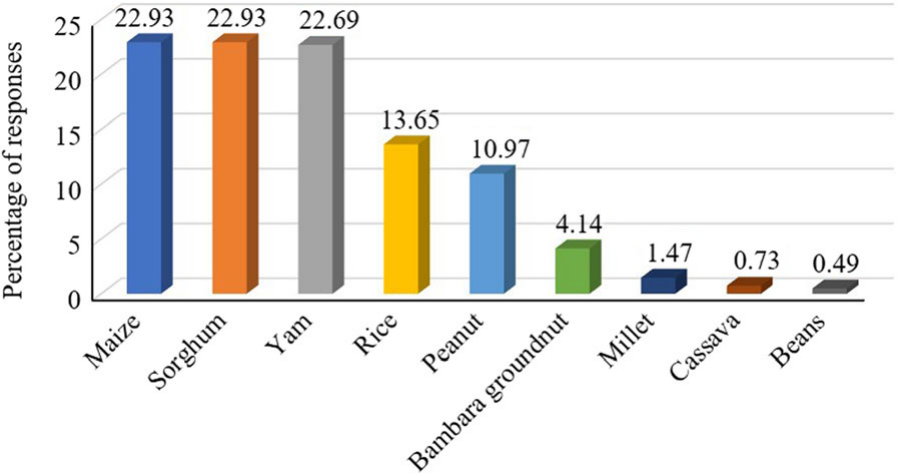
Crops reported by farmers as susceptible to termite attacks

All the surveyed farmers reported that termites, which caused an important yield loss, attacked their fields. They also reported that termites are general feeders which attack many crop species. Eight crops were listed by farmers as most susceptible to termites (Fig. 2). Among them, maize (22.93% of responses), sorghum (22.93% of responses), and yam (22.69% of responses) are considered the most susceptible. For most surveyed farmers (85.48%), the preference of termites for these crops is justified by their water content (80.37% of responses) and sweet taste (19.62% of responses). According to farmers, among factors favouring the abundance of termites in crops fields, drought (94.73% of responses) was the most important. Cow dung (2.1% of responses), chicken droppings (2.1% of responses), and weeds (1.05% of responses) were also revealed by some farmers as factors favouring the abundance of termites in farms. All termite species are considered as pests by a minority of farmers (3.19%). However, most farmers (96.81%) distinguished termite pests from non-pest species, based on several characteristics. For those farmers, termite pest species do not have winged forms (97.82% of responses), they have big heads and big mandibles (1.08% of responses), and construct termite mounds (1.08% of responses).

### Vernacular nomenclature and taxonomy of termites’ pest species

**Fig. 3:**
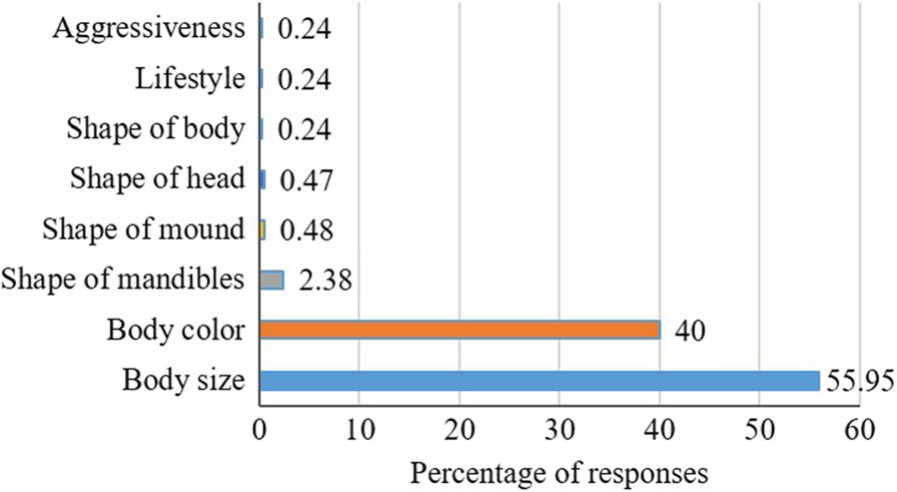
Traits used by farmers to distinguish termite pest species

The generic names of termite varied through the ethnic groups of the study area (Table 2). All the names given to termite pest species had a meaning (Table 3). The names assigned to termite pest species corresponded mainly to morphological aspects of termites (78.28% of responses), followed by the size and the shape of mandibles (16.48% of responses), termite caste (1.12% of responses), and cohesion of termites (4.12% of responses). Farmers’ identification of termites was principally based on size (55.95% of responses) and colour (40% of responses) of soldiers and workers (Fig. 3). Some farmers used the shape of mandibles (2.38% of responses), shape of mound (0.48% of responses), shape of head (0.47 of responses), shape of body (0.24% of responses), lifestyle (0.24% of responses), and aggressiveness (0.24% of responses) to distinguish termite pest species (Fig. 3).

**Table 2:** Generic name of termites, termite mounds, winged termites, and queen across ethnic groups of the study area

Ethnic groups	Termites	Termite mounds	Winged termites	Queen
Bariba	Tourou	Tourou	Yinmi	Toukorou
Waama	Touman	Touré	Iriri	Tera
Ditamari	Yétchouhinta	Ditour	Tipoulum-pouti	Tatoubota
Biali	Touapi	Touï	Yibi	Toukoué
M’bermin	Ditouré	Outougo	Nsamin	Ditoubiri
Peulh	Mohi	Touwé	Yobidji	Mohiya

**Table 3:** Meaning of termite vernacular names across ethnic groups of the study area

Criteria of denomination	Percentage of responses	Vernacular names (ethnic group)	Meaning of the vernacular name
Morphological aspect (colour and size)	78.28	Tounidé (Waama), Toukouéma (Waama),	Big termite
		Toumégan (Waama), Toubarma (Waama)	Small termite
		Ditouré (M’bermin), Dikpéri (Ditamari), Touap-tikanda (Biali),	Large red-bodied termite
		Toukouba (Bariba), Mankotobi (Ditamari), Ntoubomin (M’bermin), Nsomin (M’bermin), Mohidamédji (Peulh), Touapopoué (Biali),	Small white-bodied termite
		Mambotoumien (Ditamari), Dikoutori (M’bermin), Toukourokou or Gotourou (Bariba), Mohibodédji (Peulh), Touapiyotouhinsi (Biali), Kouba (Bariba)	Small red-bodied termite
		Itoubouo (Ditamari)	Termite with white body and red head
Size and shape of mandible	16.48	Ditour (Ditamari), Toubanga (Bariba), Gangaré (Peulh),	Big termite with large mandibles
Termites caste	1.12	Atoubi (M’bermi-n)	Child of the mother
Cohesion of termites	4.12	Dibi (Ditamari)	Termites that evolve together

### Diversity of termite pests

**Fig. 4:**
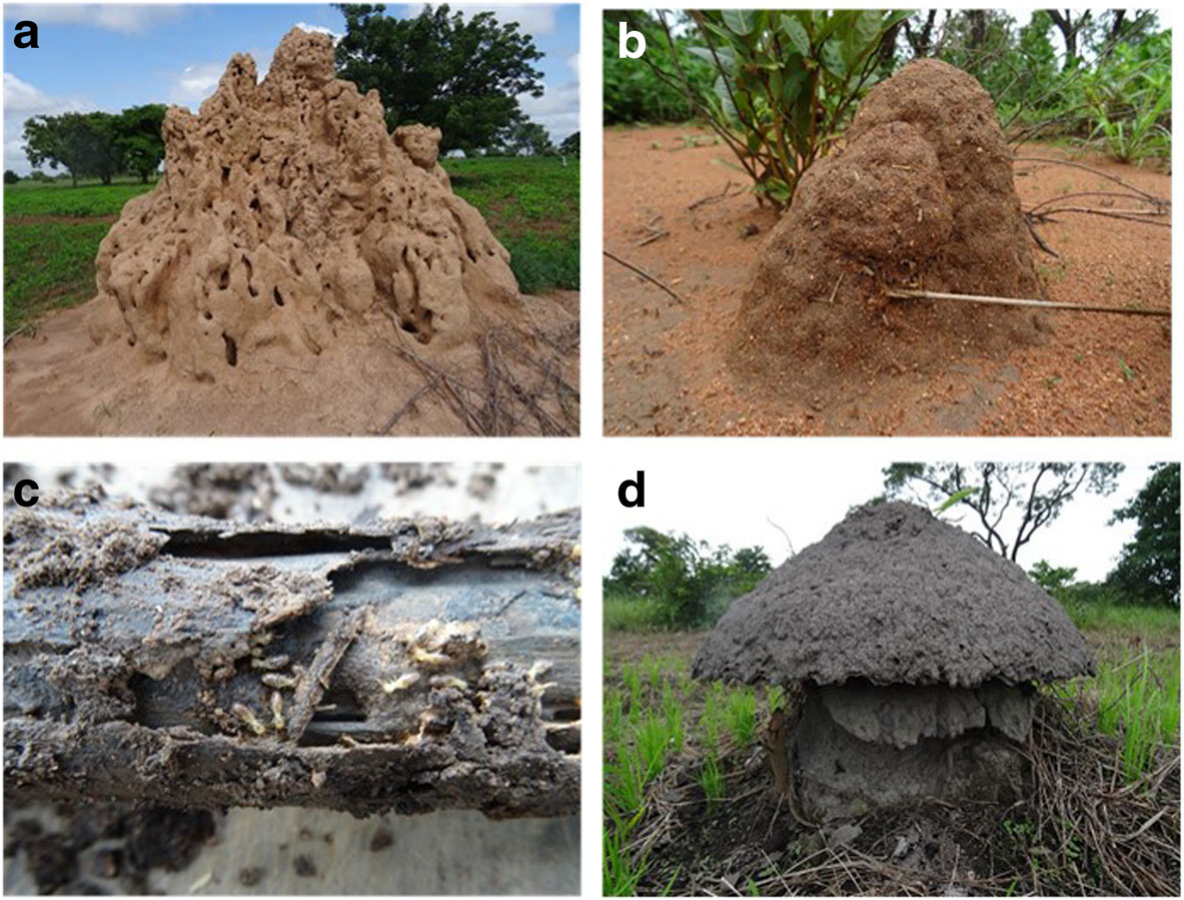
The four types of mounds of termite pests recognized by farmers. **a** Big termite mounds. **b** Small termite mounds. **c** Wood. **d** Cap-shaped termite mounds

**Fig. 5:**
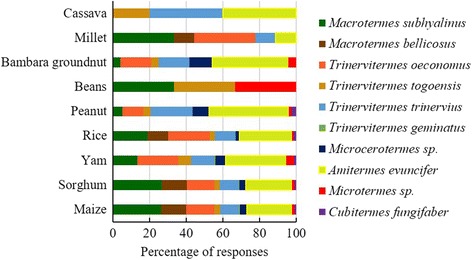
Farmers’ perception of the proportion of termite pest species which attack major crops in the study area

In the study area, between two and four species of termite pests were mentioned in the local language by each surveyed farmer. In the function of the type of termite mounds, farmers classified termite pest species in four types (Table 4). According to farmers, termite pest species can nest in big or small mounds, woods, or cap-shaped mounds (Fig. 4). A total of 27 vernacular names of termite were recorded in the study area which corresponded to 10 species (Table 4). *Amitermes evuncifer* (designated by six vernacular names), *Macrotermes subhyalinus* (designated by seven vernacular names), *Trinervitermes oeconomus* (designated by four vernacular names), and *Macrotermes bellicosus* (designated by two vernacular names) were the most mentioned termite pest species and were reported respectively by 24.24, 24.24, 15.38, and 12.69% of the respondents. *Cubitermes fungifaber* (0.77% of respondents) and *Trinervitermes geminatus* (0.38% of respondents) were the least known termite pest species (Table 4). According to farmers, maize, sorghum, yam, rice, peanut, Bambara groundnut, and millet are attacked by the majority of termite pest species (Fig. 5). However, cassava and beans were attacked only by a few species of termites (Fig. 5). Cassava was mentioned by farmers as most attacked by *Trinervitermes trinervius*, *Trinervitermes togoensis*, and *A*. *evuncifer*. In general, *M*. *subhyalinus* and *A*. *evuncifer* were the most frequently recorded as pests of many crops in the study area (Fig. 5).

**Table 4:** Farmers’ classification of termites in the function of the type of mounds and termite pest species identified by farmers (*n* = 94)

Types of nest	Vernacular name (sociolinguistic group)	Scientific name	Percentage of responses
Big termite mounds	Touap-tikanda (Biali), Toukouéma (Waama), Gangaré (Peulh), Ditouré (M’bermin), Atoubi (M’bermin), Tounidé (Waama), Dikpéri (Ditamari)	*Macrotermes subhyalinus* (Rambur, 1842)	24.24
	Toubanga (Bariba), Ditour (Ditamari),	*Macrotermes bellicosus* (Smeathman, 1781)	12.69
Small termite mounds	Touapiyotouhinsi (Biali), Toubarma (Waama), Mohibodédji (Peulh), Dikoutor (Ditamari)	*Trinervitermes oeconomus* (Trägårdh)	15.38
	Dikoutori (M’bermin), Toumégan (Waama)	*Trinervitermes togoensis* (Sjöstedt)	6.92
	Toukourokou or Gotourou (Bariba)	*Trinervitermes trinervius* (Rambur)	9.23
	Itoubouo (Ditamari)	*Trinervitermes geminatus* (Wasmann)	0.38
	Mambotoumien (Ditamari)	*Microcerotermes* sp.	3.46
Wood, straw roof, plant debris, dead leaves	Touapopoué (Biali), Mohidamédji (Peulh), Toukouba (Bariba), Mankotobi (Ditamari), Ntoubomin (M’bermin), Dibi (Ditamari)	*Amitermes evuncifer* (Silvestri)	24.24
	Nsomin (M’bermin)	*Microtermes* sp.	2.69
Cap-shaped termite mounds	Kouba (Bariba)	*Cubitermes fungifaber* (Sjöstedt)	0.77

### Termite management practices

To control termite pests, most of the farmers interviewed (54.26%) destroy termite mounds in the fields. All of them notified that only small termite mounds that prevent plowing in the fields are destroyed. The remaining farmers (45.74%) do not destroy termite mounds because of the rapid reconstruction of termite mounds by termites. Utilization of salt and chemical insecticides such as Sofagrain was mentioned by some farmers (4.25%) to reduce losses due to termites during storage. The lack of efficient pesticides for the control of termites (89.52% of responses), the toxicity of chemical to humans and animals consuming treated termites (8.57% of responses), and the lack of control methods recommended by government structures (1.91% of responses) were the main constraints related to the management of termite pests.

### Indigenous use of termite mound soil

**Fig. 6:**
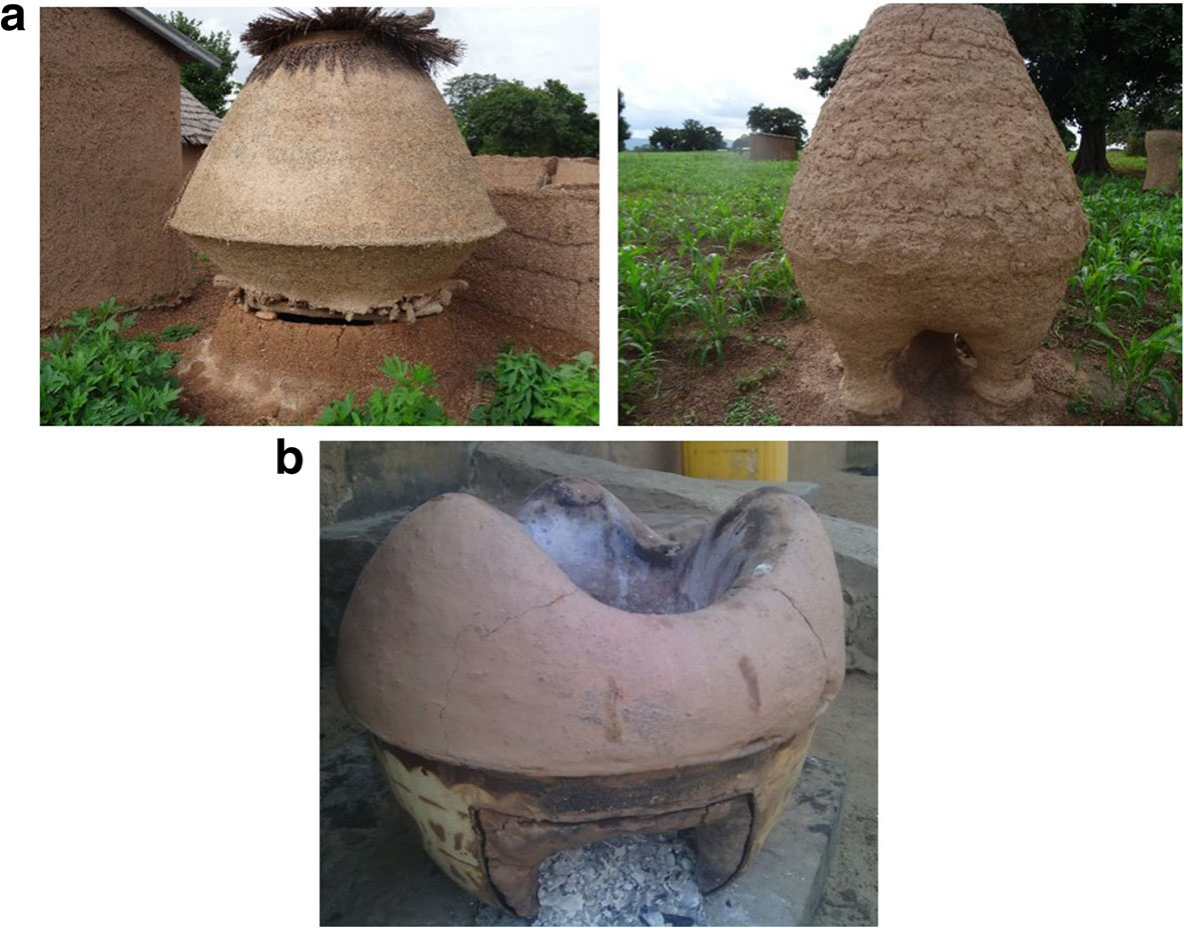
Examples of traditional granaries and furnace constructed with termite mound soil. **a** Granaries. **b** Traditional furnace

Although most interviewed farmers (91.49%) are aware that farms containing termite mounds are very fertile, none of them use termite mound soil as fertilizer on the farm. However, all interviewed farmers use termite mound soil for the construction of granaries (95.84% of responses), traditional furnaces (2.08% of responses), and houses (2.08% of responses) (Fig. 6). Farmers said that they use termite mound soil to build these structures because of its strength (87.13% of responses), its waterproofness (10.89% of responses), and its hardness (1.98% of responses). Termite mound soil is also used by some farmers (46.81%) to treat various diseases (Table 5). In Biali, M’bermin, and Ditamari ethnic groups, termite mound soil is used as plaster to support broken limbs in humans and animals (63.64% of responses) (Table 5), while in Waama, M’bermin, and Ditamari ethnic groups, termite mound soil is used as a therapeutic resource for the treatment of umbilical dermatoses (20% of responses), squirrel bites (10.91% of responses), and inflammation of the parotid glands (mumps) (5.45% of responses). Some farmers (62.5%) of Biali, M’bermin, Ditamari, Waama, and Bariba ethnic groups also use termite mound soil in traditional rituals to drive out evil spirits (90.77% of responses) and attract fortunes (6.15% of responses) and for scarification of children during the return ceremonies of their father’s maternal family (3.08% of responses). In all ethnic groups, it is especially the mound soil of *Trinervitermes oeconomus* and *Macrotermes subhyalinus* that is commonly used for these rituals.

**Table 5:** Therapeutic usages of termite mound soil by ethnic groups of Atacora department

Treated diseases	Ethnic groups	Termite mounds of species	Mode of usage
Broken limbs in humans and animals	Biali, M’bermin, Ditamari	*Macrotermes subhyalinus Macrotermes bellicosus*	Heat the soil of termite mounds with water and massage the broken limb with the obtained decoction or use it as plaster
Umbilical dermatoses	Waama	*Macrotermes subhyalinus Trinervitermes oeconomus*	Crush the soil of the termite mound, mix with water, and apply externally the obtained paste around the navel
Squirrel bites	M’bermin	*Macrotermes subhyalinus*	Dig the large termite mounds and introduce the part bitten by the squirrel in and close the termite mound. The soldiers will bite the inserted part and thus will neutralize rabies transmitted by the squirrel
Mumps	Ditamari	*Macrotermes bellicosus*	Mix the soil of termite mounds with water and smear the obtained dough on the cheeks

### Indigenous use of termites

**Fig. 7:**
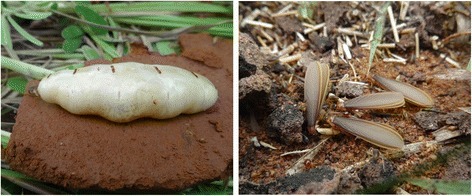
Queen and winged termites consumed by farmers in the study area

**Fig. 8:**
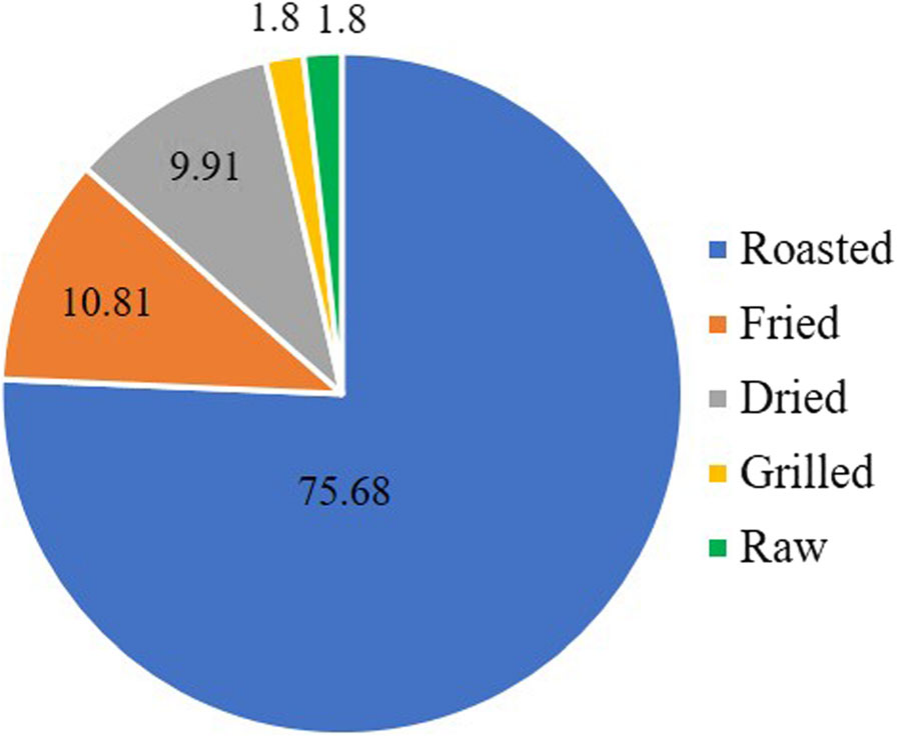
Different modes of consumption of termites in the study area

**Fig. 9:**
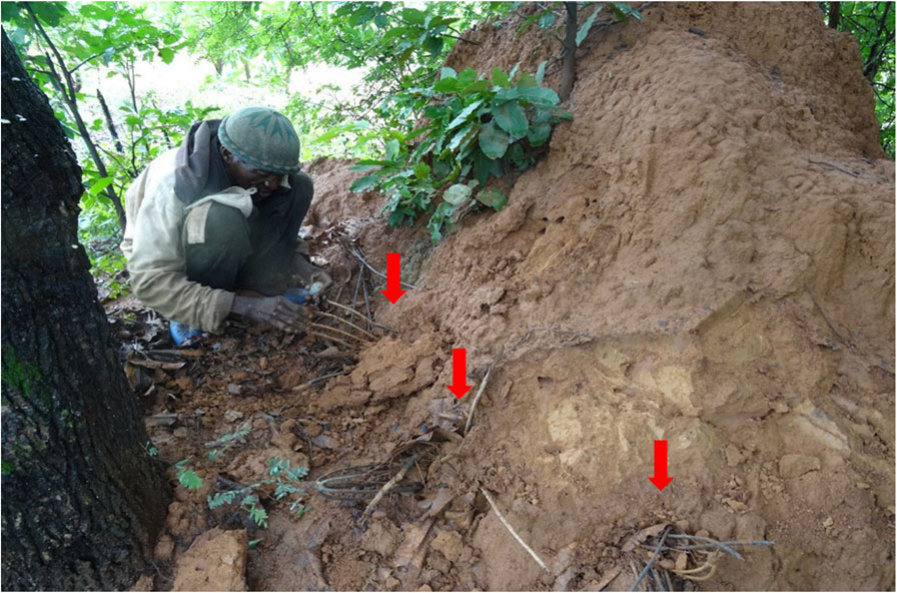
The trapping of termites for the feeding of poultry by introduction of leafy tree branches in termite mounds

In the study area, some Peulh, Ditamari, and Bariba ethnic groups (11.70% of farmers) do not eat termites, whereas most of surveyed farmers (88.30% of farmers) consume the winged termites (58.22% of responses) and queen (41.78% of responses) of some species of the subfamily Macrotermitinae whose *M*. *subhyalinus* and *M*. *bellicosus* are both considered by farmers as pests (Fig. 7). Farmers only consume winged termites and the queen of the genus *Macrotermes* because of their big size (77.06% of responses), fat (11.01% of responses), good smell (5.50% of responses), and easiness to be collected (4.59% of responses), but also by cultural heritage (0.92% of responses) and spiritual protection (0.92% of responses). In the study area, termites are being prepared for consumption in various recipes (Fig. 8). Most farmers, of all ethnic groups, consume roasted termites (75.68% of responses). Termites can be also consumed fried (10.81% of responses) by farmers of the Waama and Biali ethnic groups; dried (9.91% of responses) by farmers of the Ditamari, Waama, and Bariba ethnic groups; grilled (1.8% of responses) and raw (1.8% of responses) by farmers of the Biali and Waama ethnic groups respectively. According to farmers, termite collection is done during the rainy season either by trapping the adults with a bowl of water under a light source (58.33% of responses) or by breaking the termite mounds after the rain to recover the queen (41.67% of responses). All the surveyed farmers use workers of some termite species in poultry feeding. The trapping of termites for the feeding of poultry is done by introducing leafy tree branches (especially those of *Parkia biglobosa* (Jacq.) R.Br. ex G. Don) through a hole drilled in the termite mound (Fig. 9). The leafy branches are removed from the termite mound at a particular time usually between 30 min to 2 h, and the termites attached to the foliage are recovered in a basin. Only a few farmers (7.45%) rear termites in cow dung mixed with wood debris.

## Discussion

In the study area, termites are considered as pests for many crops. The crops mentioned by the farmers as attacked by termites have also been reported by Sileshi et al. [39] in Zambia, Maayiem et al. [40] in Ghana, and Orikiriza et al. [26] in Uganda. Many studies have also shown that maize [25, 40–43] and yam [16, 40] are the food crops most susceptible to termite attacks. It thus becomes a clear priority to evaluate the diversity and abundance of termites in maize and yam fields and to develop adequate strategies for the protection of these crops. Contrary to the reports of farmers in the study area, which mentioned sorghum as one of the major susceptible crops to termites, Maayiem et al. [40] in Ghana reported no incidence of termite attacks in sorghum fields during their study. This confirmed the claim that sorghum is protected from termite damage, perhaps due to its role as a reservoir of termite predatory ants [42]. It is apparently important to assess the impact and damage caused by termites in sorghum fields in the study area to confirm farmers’ claims.

Farmers involved in this study could identify 10 termite pest species using their indigenous names; however, there exist some farmers who lack this knowledge, for example, farmers from the central rift valley of Ethiopia [23] are not aware of the existence of different species of termites. This is also the case of Ugandan farmers of Toronto district [44] and Nakasongola district [26], which identified respectively 14 and 9 termite pest species with distinct indigenous names. Similarly, farmers in Central Benin [16], in Ghana [45], and in Uganda [26, 44] identified termite pest species principally based on their colour and size. Such indigenous taxonomic skills could be vital for communication between researchers, extension agents, educators, and farmers for termite management programs [26].

Like those in Central Benin [16], the farmers in this study recognized that several factors favoured the pest status of termites. The study reveals that termites are most damaging to crops during the dry season. Our literature review [20, 25, 39, 44] figures out similar findings. Similarly, some studies in Zambia [46] and in India [47] point out that weeds favour the abundance of termites in agricultural farms. Some farmers said that fields fertilized with cow dung are the most attacked by termites. This assertion was confirmed by Karbo et al. [48], which notified that farmers in Ghana consider cow dung obtained in the dry season as the most suitable bait for trapping termites. Moreover, Ferrar and Watson [49] have shown that termites play a crucial role in removing dry dung pads from Australian pastures, while Sileshi et al. [25] in Uganda, Malaret and Ngoru [50] in Kenya, and Banjo et al. [51] in Nigeria have reported that farmers use cow dung to control termites. It is important to conduct a pilot research to know how attractive or repulsive cow dung is to termites. In addition, farmers’ perceptions on chicken droppings favouring the attack of termites on agricultural crops need to be verified.

Results of this study showed that *A*. *evuncifer*, *M*. *subhyalinus*, *T*. *oeconomus*, and *M*. *bellicosus* are the most damaging termite species. This result is not surprising because *A*. *evuncifer* is the most common and harmful termite pest species to cassava [14], palm oil [52], rice [10], yam [14, 16], and sugarcane [44]. *Macrotermes bellicosus* and *M*. *subhyalinus* are known as the most important termite pests, causing considerable damage to agricultural crops [44, 53]. *Trinervitermes oeconomus* is regarded as a significant pest of yam [16]. Among these termite pest species cited by farmers, *Microtermes* sp. is recognized as the most damaging species to groundnut [54] and maize root systems [10]; *Microcerotermes* sp. and *T*. *trinervius* causing damage to cassava cuttings [1]; and *T*. *geminatus*, *T*. *togoensis*, and *Cubitermes fungifaber* as pests of mangoes [19]. In the study area, farmers identified several species of termites as associated with each of major crops.

Most of the farmers in the study area systematically and deliberately demolish the termite mounds near the crop farms to control the pests. This control method was also practised by Ugandan farmers [44, 55]. Knowing that termite species play a beneficial role in the promotion of essential ecological processes [25], it is therefore important to increase farmers’ knowledge on these roles in the farm ecosystem. Utilization of salt to control termites in the study area has been also reported in Ghana during a similar study [45]. Despite the effectiveness of salt to dehydrate insects [56], it is expensive for farmers to purchase the quantities to be used across the land of the plot [40]. Very few farmers use chemical pesticides to control termites because most of them are inefficient. As mentioned by farmers, it is important to develop termite control strategies that will be disseminated through government structures.

Although all farmers in the study area have attested that lands containing termite mounds are fertile, none of them fertilizes the crop fields with the soil of termite mounds. However, the soil of termite mounds is used as fertilizer by some farmers in Ethiopia [23], Laos [57], Uganda [58], Zambia, Zimbabwe, Tanzania, Niger, and Sierra Leone [25]. Indeed, the soil of termite mounds is usually rich in minerals, such as calcium, magnesium, potassium, sodium, and available phosphorus [59, 60]. Studies conducted by Miura et al. [61] and Noble et al. [62] in Northeast Thailand show higher crop productivity at the site of levelled termite mounds. Similarly, to farmers of this study, soil from termite mounds is also principally used for granary construction in Ghana [45]. Indeed, the treated termite mound clay silos demonstrated great potential for reducing temperature fluctuations and maintaining stored grain quality [63]. As farmers in this study also mentioned, the soil of termite mounds is also used by Ghanaian farmers as medicines for the treatment of various diseases that affect humans [45].

Termites are widely consumed by people from all over the world because they provide the relevant amount of organic nutrients [64]. As already mentioned by farmers, edible termites in Africa are represented by the *Macrotermes* species [65]. Winged termites of the genus *Macrotermes* are also mostly consumed by people in Nigeria [66, 67], Kenya [68], South Africa [69], and India [70]. Similarly, to the Nigerian people [71], winged termites are mostly roasted and relished as a snack by people living the traditional lifestyle. In the study area, no utilization of termites in traditional medicine was recorded, while the evidence of antibiotic [72, 73], antimicrobial [74], antifungal [75], and antibacterial [75, 76] properties of some termite species have been reported. Therefore, the consumption of termites by the farmers in the study area must be promoted because the species of termites consumed can be considered as nutraceuticals. The high-fat content of termites justifies their widespread utilization for poultry feeding by farmers in West Africa [77]. However, few farmers rear termites that are collected from the mounds. In view of the difficulty to find adequate quantities of termites for farm animals, efficient methods of rearing termites based on fibrous and humidified waste or crop residues placed in clay pots or baskets, which are then inverted and placed on small termite nests as described by Vorsters et al. [78], should be disseminated to farmers.

## Conclusion

This study showed that termites are important pests of many crops with maize, sorghum, and yam as being the most susceptible. According to farmers, *A*. *evuncifer*, *M*. *subhyalinus*, *T*. *oeconomus*, and *M*. *bellicosus* are the most damaging termite pest species. It is important to evaluate the diversity, abundance, impact, and damage of termite pests on crop production for their better management. The documentation of vernacular nomenclature and taxonomy of termites’ pest species is expected to help the communication between researchers and farmers. The role of cow dung and chicken droppings as factors favouring termite attacks on agricultural crops need to be verified. Many strategies have been developed by farmers to reduce the attack of termites; among them, the destruction of termite mounds in the fields was the most important. So, it is urgent to sensitize farmers on the ecosystemic importance of these social insects but also to develop strategies for pest management permitting the conservation of non-pest termite species. This study also revealed several utilizations of termites and the soil of termite mounds by farmers showing their potential for being used as food, building materials, and new medicinal sources.

## Abbreviations

G. Don: Georges Don
(Jacq.): Jacquin
R.Br.: Robert Brown
